# Study on the Mechanism of Curcumin Regulating Lung Injury Induced by Outdoor Fine Particulate Matter (PM2.5)

**DOI:** 10.1155/2019/8613523

**Published:** 2019-08-21

**Authors:** Kunlun Huang, ChanMei Shi, JingQi Min, Laifu Li, Tao Zhu, Huapeng Yu, Huojin Deng

**Affiliations:** ^1^Department of Pulmonary and Critical Care Medicine, Zhujiang Hospital, Southern Medical University, Guangzhou 510280, China; ^2^Department of Respiratory Medicine, Second Affiliated Hospital of Chongqing Medical University, Chongqing 400010, China

## Abstract

**Background:**

Epidemiological studies have shown that exposure to PM induces oxidative stress, leading to a variety of health problems. In particular, PM2.5 contains a lot of substances harmful to the human body and penetrates into the lungs to induce lung injury. At the same time, there is increasing evidence that oxidative stress also affects the severity of lung injury. However, there is still no good way to reduce or eliminate these hazards. In the future, more experimental research is needed to further confirm the mechanisms of these hazards and formulate effective preventive measures and treatment plans for their hazard mechanisms. Curcumin has been reported to reduce oxidative stress and inflammatory damage and protect organs.

**Objective:**

To investigate whether curcumin can play a protective role against PM2.5-induced oxidative stress and inflammatory damage by inducing expression of the HO-1/CO/P38 MAPK pathway.

**Methods:**

In this experiment, PM2.5 was dropped into the trachea to establish a lung injury model in mice. 28 SPF-grade male Kunming mice were randomly divided into 4 groups: normal control group, saline control group, PM2.5 treatment group, and curcumin intervention group. Albumin (ALB), lactate dehydrogenase (LDH), and alkaline phosphatase (ALP) were measured in alveolar lavage fluid (BALF) to assess lung tissue damage. Colorimetric detection of oxidative stress indicators such as MDA, GSH-PX, T-AOC, and CAT in the lung tissue was performed. The levels of IL-6 and TNF-*α* in the lung tissue were determined by ELISA. Histopathological examination was used for the assessment of alveolar epithelial damage. The protein expression of the HO-1/P38 MAPK pathway in the lung tissue was determined by Western blot and immunohistochemistry. Endogenous CO was detected by spectrophotometry. The results showed that the expression of the HO-1/CO/P38 MAPK protein in the lung tissue was significantly increased in the curcumin intervention group compared with the PM2.5 treatment group, and it was statistically significant (*P* < 0.05). Compared with the PM2.5 treatment group, the curcumin intervention group can reduce the amount of ALB, LDH, and ALP in BALF; reduce the levels of MDA, IL-1, and TNF-*α* in the lung tissue; and improve GSH-PX, T-AOC, and CAT levels, but there is no statistical difference (*P* > 0.05).

**Conclusion:**

We found that PM2.5 can cause lung damage through oxidative stress and inflammatory responses. Oxidative stress and inflammatory responses increase the expression of HO-1/CO/P38 MAPK. The intervention of curcumin can further increase the expression of HO-1/CO/P38 MAPK.

## 1. Background

Air pollution poses a huge environmental risk to health. According to the Global Burden of Disease report, outdoor fine particulate matter (PM2.5) exposure is the fifth largest risk factor for death in the world, resulting in 4.2 million deaths and more than 100 million disability-adjusted life-year losses. The World Health Organization attributed 3.8 million deaths to indoor air pollution [1]. Air pollution poses a direct and major threat to human health by inducing serious respiratory diseases such as chronic obstructive pulmonary disease (COPD) and asthma [2, 3] and can also be used as a trigger for various forms of interstitial lung disease and lung cancer. The severity of air pollution was measured by measuring airborne particles (PM) [4]. The PM consists of fine particles and coarse particles: the fine particles have an aerodynamic diameter of ≤2.5 *μ*m, which is called PM2.5, and the coarse particles have an aerodynamic diameter of ≤10 *μ*m, which is called PM10. The two types of PM are mainly from vehicles, industrial waste gas, and household sources [5]. It is well known that both PM2.5 and PM10 can cause severe respiratory damage [6]. Airborne particulate pollutants, especially PM2.5, contain a large amount of substances harmful to humans [7, 8], deep into the lungs to induce oxidative stress, which can lead to lung injury [9–12]. Studies have shown that PM2.5 is also a risk factor for chronic obstructive pulmonary disease (COPD), aggravating the degree of lung injury in patients with COPD [11]. After exposure to PM2.5, it can promote the release of various inflammatory factors in the lungs, produce a large number of oxygen free radicals, destroy the body's oxidation/antioxidant balance, and cause damage to the respiratory system and other systems. The role of oxidative stress mechanisms in lung diseases has received increasing attention. The imbalance of oxidation/antioxidation is the key to instability in pulmonary disease [13–15], the reduction or even absence of antioxidants enhances the oxidative stress response of the body, and the oxygen free radicals increase accordingly. After acting on the bronchial epithelial cells, the cells are overoxidized, and the membrane structure, mitochondrial membrane, and other membrane-like structures increase the permeability of epithelium [16], simultaneously causing DNA strand breaks during peroxidation [17] and cell death.

Heme oxygenase 1 (HO-1) is a protease of mammalian tissue microsomes, a rate-limiting enzyme for heme metabolism and a rate-limiting and key enzyme for endogenous carbon monoxide (CO) synthesis, which can decompose heme into bilirubin, CO, and iron, all of which are important biological effector molecules [16]. HO-1 exerts physiological functions such as antioxidation, anti-inflammation, and antiapoptosis through these effector molecules in vivo; bilirubin is an effective antioxidant in vivo, which has the function of scavenging free radicals, and participates in maintaining the balance of oxidation and antioxidant mechanisms in vivo. CO can exert antiapoptotic and antiproliferative effects through the p38 MAPK signaling pathway [18]. It can also combine the ferrous ions of the heme group in guanylate cyclase to catalyze the production of cGMP, thereby exerting various physiological effects, etc. HO-1 stimulates cells to produce ferritin, which not only prevents iron-mediated cytotoxicity but also inhibits apoptosis [19]. As a stress protein, HO-1 acts as an adaptive and protective response to noxious stimuli and is the most frequently induced protein. Recent studies have confirmed that the induction of HO-1 is an important part of the body's antioxidant damage and adaptive changes [20, 21].

Curcumin is a fat-soluble polyphenolic pigment extracted from the rhizome of the gingeraceae turmeric. Many studies have shown that [22–25] curcumin has obvious biological effects such as antioxidative stress, anti-inflammation, and antiapoptosis and plays a role in multiple molecular targets of multiple pathways, thus protecting multiple organ functions. The results show that [26, 27] curcumin has a good alleviating effect on lung injury caused by various causes, and the mechanism of action is more complicated.

In this experiment, an early intervention method of curcumin was used to establish a mouse lung injury model based on PM2.5-induced lung injury-related experiments [28], to investigate whether curcumin can alleviate lung injury through the HO-1/CO/P38 MAPK pathway.

## 2. Materials and Method

### 2.1. Material

Curcumin ([1,7-bis(4-hydroxy-3-methoxyphenyl)-1,6-heptadiene-3,5-dione]; high purity ≥ 98.5%) is available from Sinopharm Chemical Reagent Co. Ltd.

PM2.5 is granted by the Heart Experimental Center of Zhujiang Hospital, Southern Medical University (traffic related PM2.5).

The experimental animals were 28 SPF-grade adult Kunming male mice (purchased from the Laboratory Animal Center of Southern Medical University), with a body weight of 28-32 g. They were randomly divided into the normal control group, saline control group, PM2.5 treatment group, and curcumin intervention group. All studies were reviewed and approved by the Animal Ethics Committee according to the protocol, and the mice received the same food and water with maintained temperature, humidity, and noise under the same environmental conditions. After 1 week of adaptive feeding, experiments were carried out according to the research design.

Polyformaldehyde and hematoxylin-eosin (HE) staining solution was purchased from Servicebio and operated according to the instructions.

The lactic dehydrogenase (LDH), alkaline phosphomonoesterase (ALP) and albumin (ALB) test kits were purchased from Changchun Huili Company and tested according to the instructions. The malondialdehyde (MDA) test kit, total antioxidative capacity (T-AOC) test kit, catalase (CAT) test kit, glutathione peroxidase (GSH-Px) test kit, and the endogenous CO test kit were purchased from Nanjing Jiancheng Company and tested according to the instructions.

Tumor necrosis factor- (TNF-) *α* and cytokine interleukin-6 (IL-6) were purchased from Thermo Fisher Scientific Company and tested according to the instructions.

The kits for hemoglobin oxygenase 1 (HO-1) and P38 MAPK were purchased from Servicebio and tested according to the instructions.

### 2.2. Experimental Procedure

28 SPF Kunming male mice were randomly divided into groups A–D, group A (normal control group, 6 mice), group B (normal saline control group, 6 mice), group C (PM2.5 treatment group, 8 mice), and group D (curcumin intervention group, 8 mice). Group A received normal feeding. Group B was intraperitoneally injected with normal saline (NS) (0.3 ml/time, 1/d for 7 days) but, on the 8th day, received intratracheal instillation of NS (0.2 ml, 1/d for 3 consecutive days). Group C received intraperitoneal injection of normal saline (0.3 ml/time, 1/d, a total of 7 days) but, on the 8th day, began receiving intratracheal instillation of PM2.5 [29] (20 mg/kg, 0.2 ml, 1/d for 3 consecutive days). Group D was injected intraperitoneally (normal saline+dimethyl sulfoxide (DMSO)+curcumin, 100 mg/kg) [30] (0.3 ml/time, 1/d, continuous injection for 7 days) but, on the 8th day, received intratracheal instillation of PM2.5 (20 mg/kg, 0.2 ml, 1/d for 3 consecutive days). The alveolar lavage fluid and lung tissue of A-D mice were taken on the 11th day.

In the morphological observation of the lung tissue, the lungs of the upper lobe of the left lung of each group were fixed with 4% polyformaldehyde and infiltrated, dehydrated, and paraffin-coated sections (thickness 6 *μ*m); stained with HE; and observed under light microscope.

The HE staining and pathological grading criteria for the lung tissue of mice were established.

The criteria are based on the pathological grading standards of Szarka et al. [31]; the lung injury status is divided into a total of 6 levels (0–5) (as shown in Table 1).

## 3. Statistical Analysis

The analysis was performed using SPSS19.0 statistical software; the experimental results were expressed as *x* ± *s*. Comparisons between groups were performed by the one-way analysis of variance and LSD test of pairwise comparisons. The multiple independent sample nonparametric tests were used for ranked data. *P* < 0.05 was considered to be a statistically significant difference.

### 3.1. Pathological Changes in Lung Tissue


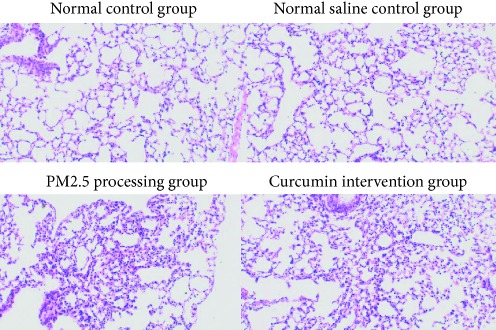


The pathological changes of the lung tissue were observed under a light microscope. The PM2.5 treatment group and the curcumin intervention group had obvious inflammatory lesions compared with the two control groups, mainly showing monocyte infiltration and lymphocyte proliferation, mucosal damage, blood vessels, bleeding, etc. But there was no significant difference in pathological changes under the light microscope between the PM2.5 treatment group and the curcumin intervention group.

### 3.2. PM2.5 Cytotoxicity to Lung Tissue

**Figure 1:**
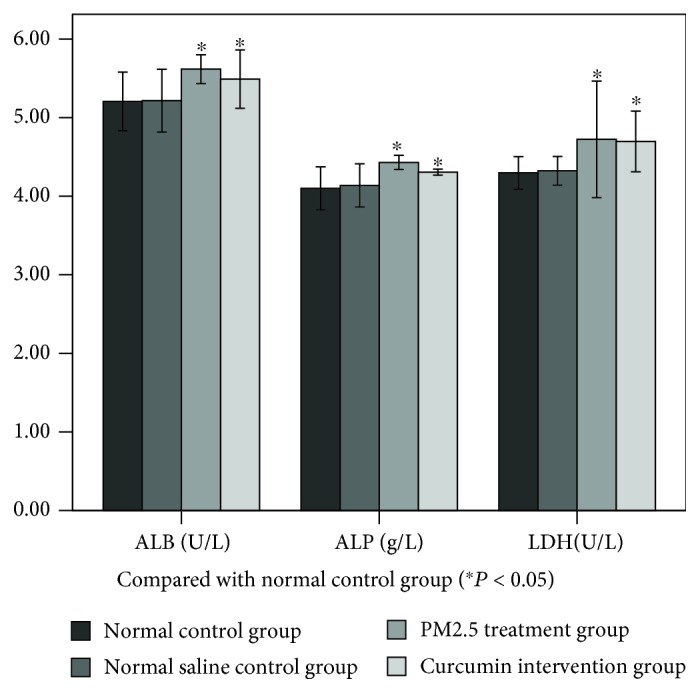
Effects of the PM2.5 on ALP, LDH, and ALB in bronchoalveolar lavage fluid (BALF).

The results are shown in Figure 1. There were no significant differences in the LDH, ALP, and ALB indices in lung lavage fluid. There was no significant difference between the normal control group and the saline control group. The difference between the PM2.5 treatment group and the saline control group was statistically significant (*P* < 0.05). The difference between the curcumin intervention group and the saline control group was statistically significant (*P* < 0.05). However, there was no significant difference between the PM2.5 treatment group and the curcumin intervention group.

### 3.3. Changes in Oxidative Stress and Inflammatory Markers in Lung Tissue

**Figure 2:**
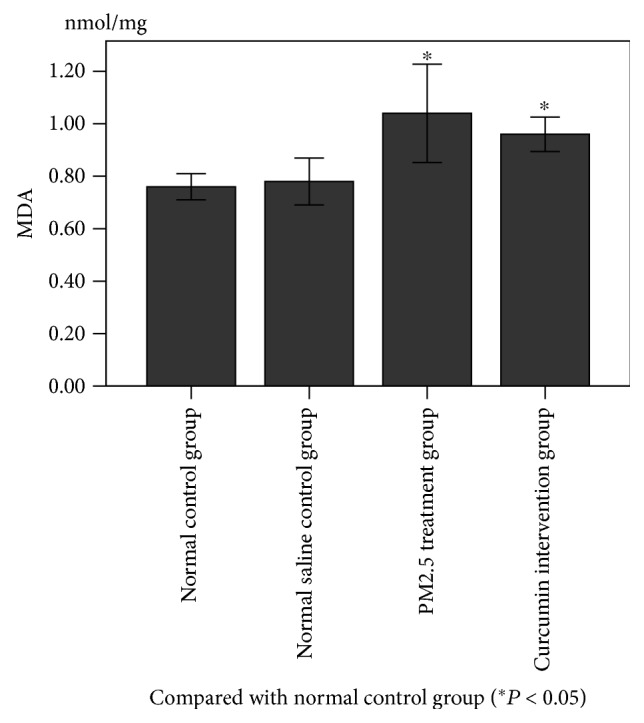


**Figure 3:**
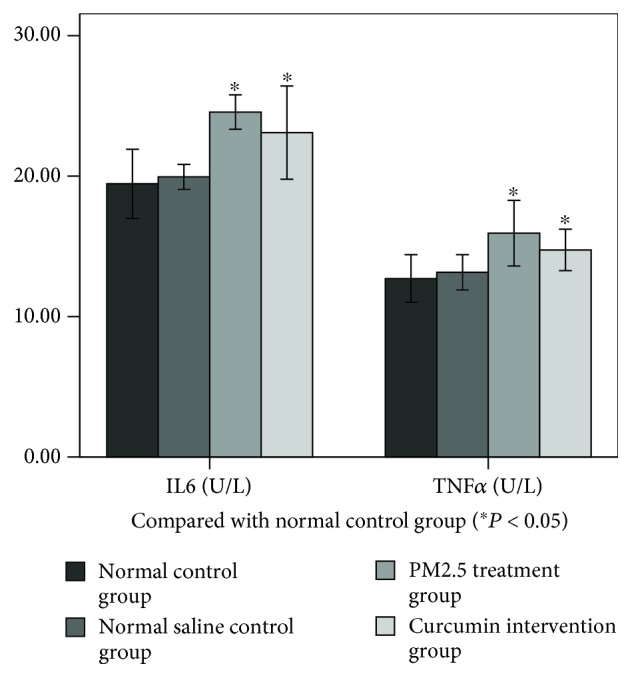


As can be seen from Figures 2 and 3, the levels of MDA, TNF-*α*, and IL-6 in the lung tissue were measured. There was no significant difference between the normal control group and normal saline control group. The difference between the PM2.5 treatment group and normal saline control group was statistically significant (*P* < 0.05). The difference between the curcumin intervention group and normal saline control group was statistically significant (*P* < 0.05). However, there was no significant difference between the PM2.5 treatment group and the curcumin intervention group.

### 3.4. Changes in Antioxidant Index in Lung Tissue

**Figure 4:**
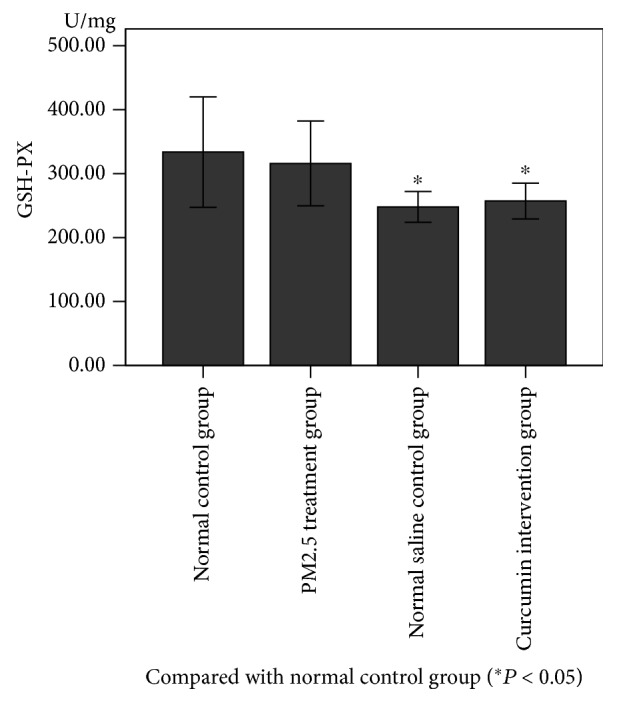


**Figure 5:**
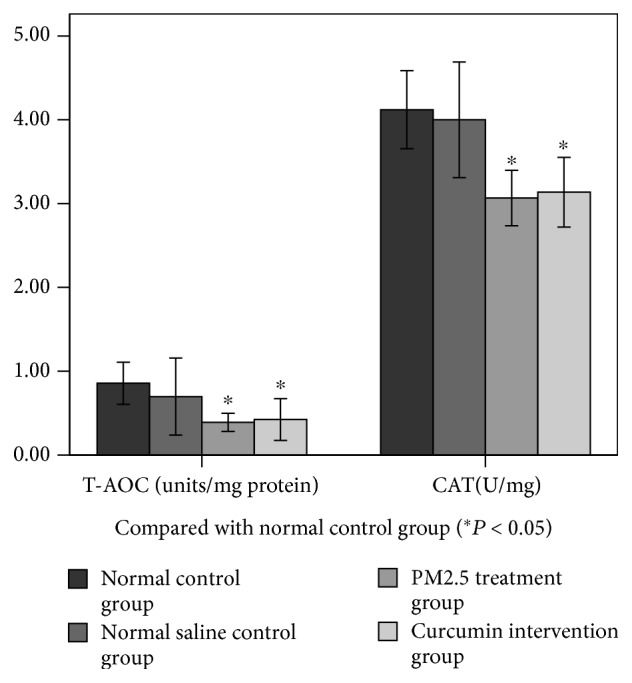


As can be seen from Figures 4 and 5, the levels of GSH-PX, T-AOC, and CAT in the lung tissue were measured. There was no significant difference between the normal control group and the saline control group. There was a significant difference between the PM2.5 treatment group and the saline control group (*P* < 0.05). There was a significant difference between the curcumin intervention group and the saline control group (*P* < 0.05). However, there was no significant difference between the PM2.5 treatment group and the curcumin intervention group.

### 3.5. Content of HO-1/CO/P38 MAPK in Lung Tissue

**Figure 6:**
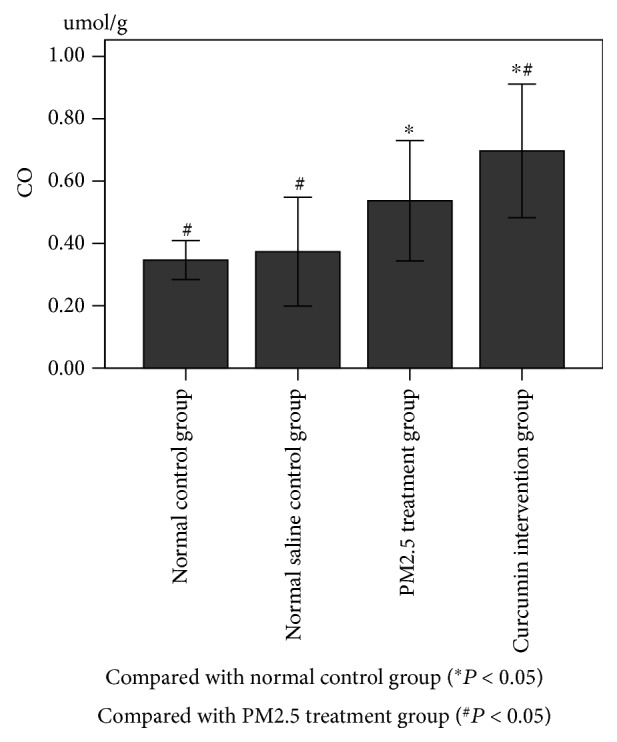


**Figure 7:**
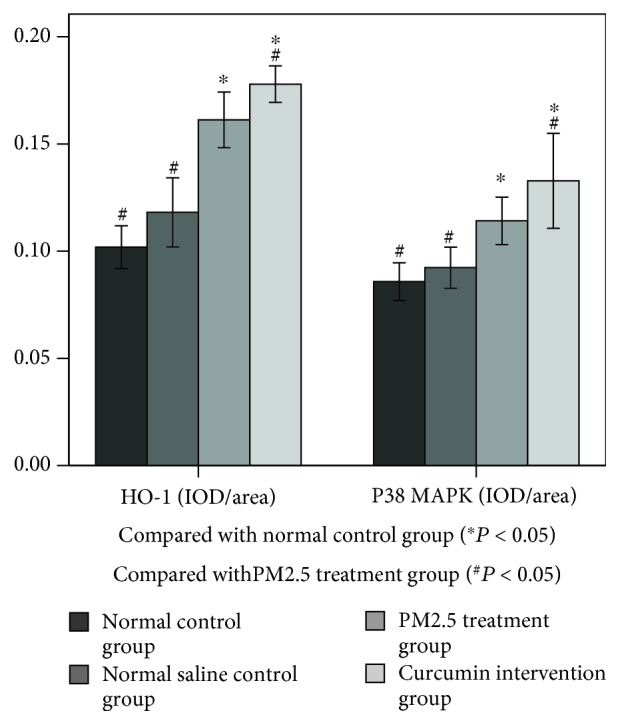


**Figure 8:**
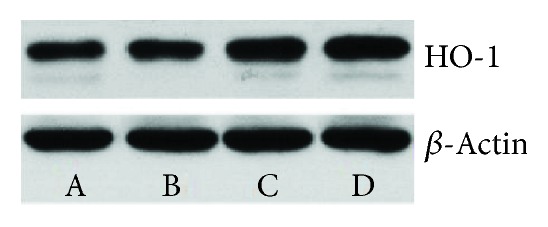
Western blot (WB).

As shown in Figures 6–8, there was no significant difference between the normal control group and the saline control group. There was a significant difference between the PM2.5 treatment group and the saline control group (*P* < 0.05). There was a significant difference between the curcumin intervention group and the saline control group (*P* < 0.05). There was also a significant difference between the PM2.5 treatment group and the curcumin intervention group (*P* < 0.05).

## 4. Discussion

After being inhaled into the lungs, PM2.5 is deposited in the lungs, mainly in the alveoli. The main mechanisms are inertial collision, precipitation, and diffusion, with precipitation and Brownian diffusion dominating [32]. In the inhaled PM2.5, it usually contains metals such as iron, copper, and zinc [33, 34]; on the one hand, the redox-active metal in PM2.5 initiates a series of cleavage reactions and the formation of free radicals, which can lead to cell membrane lipids, peroxidation, and inflammation [32]; on the other hand, PM2.5 is phagocytized by alveolar macrophages, releasing airway inflammatory mediators, mainly IL-6, IL-8, IL-13, TNF-*α*, macrophage inflammatory protein-1, granulocyte-macrophage colony-stimulating factors, etc. [35, 36]. These factors can directly lead to the occurrence of inflammatory reactions, leading to damage in the lung tissue [37]. LDH is a cytoplasmic enzyme that is released in large quantities when cell membranes are damaged or cell death is dissolved: not only can it effectively scavenge free radicals and endogenous oxidizing active substances, but it can also be a regulator of antioxidant enzymes (SOD, GSH-PX, and CAT); ALB-derived plasma exudates reflect the extent of alveolar epithelial-capillary barrier damage. ALP is mainly produced by alveolar type II cells, partly produced by neutrophils, and its activity suggests the degree of damage to alveolar type II cells and the degree of neutrophil infiltration. In this experiment, the lung injury was evaluated according to the LDH, ALB, ALP content and lung tissue section scores in the alveolar lavage fluid of mice. The PM2.5 treatment group and the curcumin intervention group were higher than the control group, suggesting that PM2.5 entered the body, causing damage to the lung parenchyma and biofilm and pathological damage such as lung tissue congestion, neutrophil infiltration, and inflammatory exudation. It shows the toxic effect of granule on the lung tissue.

Curcumin is a natural antioxidant [38, 39]: not only can it effectively scavenge free radicals and endogenous oxidizing active substances, but it can also be a regulator of antioxidant enzymes (SOD, GSH-PX, and CAT). It can also increase intracellular glutathione levels by maintaining the activity of histone acetyltransferase in monocytes [40], thereby mitigating damage during oxidative stress [39]. In recent years, it has been found that one of the core mechanisms by which curcumin exerts its protective effect is to promote the activation of the Nrf2 signaling pathway in cells and induce the expression of phase II detoxification enzymes and antioxidant enzymes, such as nicotinamide quinone oxidoreductase 1 (NQO1), *γ*-glutamylcysteine synthetase (*γ*-GCS), HO-1, and superoxide dismutase (SOD). These enzymes protect the body from active substances and some toxic substances, thereby exerting oxidative stress, which is a protective effect of injury [41, 42].

As an endogenous active enzyme, HO-1 is widely distributed in damaged organs and plays a role in protecting tissues and organs through multiple mechanisms such as antioxidation, anti-inflammation, and antiapoptosis [43]. The protective effect of HO-1 may also be related to its catalysis of heme production of bilirubin, CO scavenging free radicals, and alleviating lipid peroxidation [44–46]. Furuyama et al. [47] found that the organic extracts of diesel exhaust gas were found to induce HO-1 production, and HO-1 plays an important role in protecting cells from oxidative damage.

Heme metabolite CO has antioxidation, anti-inflammatory and antiapoptotic effects, which many scholars observe [48–50]; it is shown that CO activation of P38 MAPK is involved in its cytoprotective effect. Brouard et al. [51] believed that HO-1-derived CO activates P38 MAPK and inhibits TNF*α*-induced apoptosis of endothelial cells. Otterbein et al. [50] believed that CO binds to the heme structural unit in the P38 MAPK upstream kinase and acts as an anti-inflammator.

Exposure to PM2.5 can lead to lipid peroxidation and inflammation of the cell membrane, and there is increasing evidence that oxidative stress affects the severity of lung injury [52–54]. Lipid peroxidation is one of the main mechanisms of ROS-induced cell damage. The unsaturated fatty acids contained in the biofilm are susceptible to ROS attack and lipid peroxidation. As one of the lipid peroxidation products, MDA can reflect the degree of lipid peroxidation and the intensity of cells attacked by free radicals [55]. In this study, we confirmed the MDA level and understood the extent of membrane lipid peroxidation through the level of MDA to indirectly assess the extent of oxidative stress.

In this experiment, WB and immunohistochemistry were used to detect the expression level of HO-1 protein, and the expression of CO/P38 MAPK in downstream products was detected by immunohistochemistry. The experimental results showed that the expression of HO-1/CO/P38 MAPK was significantly enhanced under PM2.5 exposure, which suggested that the inflammatory and oxidative stress reactions induced the expression of antioxidant HO-1 and its downstream product CO/P38 MAPK. The results showed that the expression of the HO-1 protein was detected by WB and immunohistochemistry 3 days after exposure to PM2.5, and the expression of CO/P38 MAPK was detected by immunohistochemistry. The expression of HO-1/CO/P38 MAPK was significantly higher than that of the normal control group and the saline control group, suggesting that PM2.5-induced inflammation and oxidative stress caused an increase in the expression of HO-1 and its downstream product CO/P38 MAPK. It can be speculated that PM2.5 can activate the expression of HO-1/CO/P38 MAPK and resist the damage of the lung induced by PM2.5-induced oxidative stress and inflammatory response.

In the curcumin intervention group and the PM2.5 treatment group, the expression level of HO-1/CO/P38 MAPK in the curcumin intervention group was significantly higher than that in the PM2.5 intervention group, indicating that curcumin could increase HO-1/CO/P38 MAPK pathway protein expression.

In this experiment, the oxidative substances and MDA, TNF-*α*, IL-6, and other inflammatory factors in the curcumin intervention group were lower than those in the PM2.5 intervention group, but there was no statistical significance. In the curcumin intervention group, the antioxidant capacity of the curcumin intervention group was stronger than that of the PM2.5 intervention group, with no statistical significance. In the lung histopathology score, the curcumin intervention group score was lower than the PM2.5 intervention group score, but it was still not statistically significant. The results showed that the expression of the HO-1/CO/P38 MAPK pathway was increased after curcumin intervention.

It is speculated that the possible causes are as follows: (1) the experimental period is too short, and the expression of oxidative stress index and inflammatory factor has a delay. (2) The experimental sample is insufficient.

Based on the results of this experimental study, we hypothesized that the expression of HO-1/CO/P38 MAPK was increased in mice after PM2.5 exposure, but its protective effect was not sufficient to completely offset the PM2.5-induced inflammation and oxidative damage, eventually leading to lung tissue damage. Compared with the PM2.5 treatment group, the expression level of HO-1/CO/P38 MAPK was significantly increased in the curcumin intervention group, indicating that curcumin can increase the expression level of HO-1/CO/P38 MAPK. In this experiment, we found that PM2.5 can cause lung damage through oxidative stress and inflammatory responses. Oxidative stress and inflammatory responses increase the expression of HO-1/CO/P38 MAPK. The intervention of curcumin can further increase the expression of HO-1/CO/P38 MAPK. In the comparison of oxidation/antioxidation index, inflammation index, and pathological score, the correlation index of the curcumin intervention group was better than that of the PM2.5 treatment group, but it was not statistically significant. It is necessary to add experimental periods and samples in subsequent experiments to verify the role of curcumin in reducing or eliminating oxidative stress and inflammatory damage in the respiratory system through HO-1/CO/P38 MAPK.

## Figures and Tables

**Table 1:** Pathology classification standard of lung injury.

Grading	Basis
0	Alveolar wall intact without thickening, no inflammatory infiltration, no congestion
1	Slight diffuse inflammatory cell infiltration (neutrophils) in the alveolar wall, no thickening of the alveolar wall
2	Obvious and extensive inflammatory cells (neutrophils and monocytes) infiltrated, slightly thickened alveolar walls (1-2 times)
3	Severe inflammatory cell infiltration, basal thickening of alveolar wall in individual areas (2-3 times)
4	Severe inflammatory cell infiltration, thickened alveolar wall, 25-50% pulmonary consolidation
5	Severe inflammatory cell infiltration, thickened alveolar wall, >50% pulmonary consolidation

## Data Availability

The data used to support the findings of this study are available from the corresponding author upon request.
